# A Rare Coexistence of Isolated Unilateral Conjunctival Telangiectasia and Retinal Vascular Tortuosity

**DOI:** 10.1155/2020/8814961

**Published:** 2020-08-24

**Authors:** Hilal Kilinç Hekimsoy, Mehmet Ali Şekeroğlu

**Affiliations:** University of Health Sciences, Ulucanlar Eye Training and Research Hospital, Ophthalmology Clinic, Ankara, Turkey

## Abstract

A 55-year-old woman with no known systemic disorder and without any history of ocular disease, trauma, and surgery presented with a nonremitting conjunctival redness on her left eye that was existing since her childhood. On ophthalmological examination, an extremely rare coexistence of isolated unilateral bulbar conjunctival telangiectasia and ipsilateral retinal vascular tortuosity without any systemic and neuroradiological association was detected. We aimed to demonstrate this rare vascular coexistence and discuss differential diagnosis of the underlying causes.

## 1. Introduction

Conjunctival telangiectasia is a rarely encountered ocular finding which can be described as the presence of tiny dilatations of conjunctival blood vessels. It can be congenital and can accompany some systemic syndromes such as hereditary hemorrhagic telangiectasia (Rendu-Osler-Weber disease), ataxia telangiectasia, Fabry's disease, Alport syndrome, and Bloom syndrome or can be acquired especially secondary to some chronic ocular surface disorders such as ocular rosacea [1–4]. Conjunctival telangiectasia may involve one or both of the arterial and venous systems; it can occur unilaterally or bilaterally, and it can be localized or generalized.

Herein, we report an extremely rare coexistence of isolated unilateral bulbar conjunctival telangiectasia and ipsilateral retinal vascular tortuosity without any systemic or neuroradiological association and aim to discuss the differential diagnosis of the possible underlying diseases.

## 2. Case Report

A 55-year-old woman with no known systemic disorder and without any history of ocular disease, trauma, and surgery presented with a nonremitting conjunctival redness on her left eye that was existing since her childhood. Since she has never reported none of the ocular symptoms such as burning and foreign body sensation accompanying to redness in her eye, she had received several topical treatments including artificial tear drops, steroids, antibiotics, and nonsteroidal anti-inflammatory eye drops with poor response. She denied any family history and accompanying dermatological disorder or any systemic diseases such as hypertension, diabetes, and hyperlipidemia.

**Figure 1:**
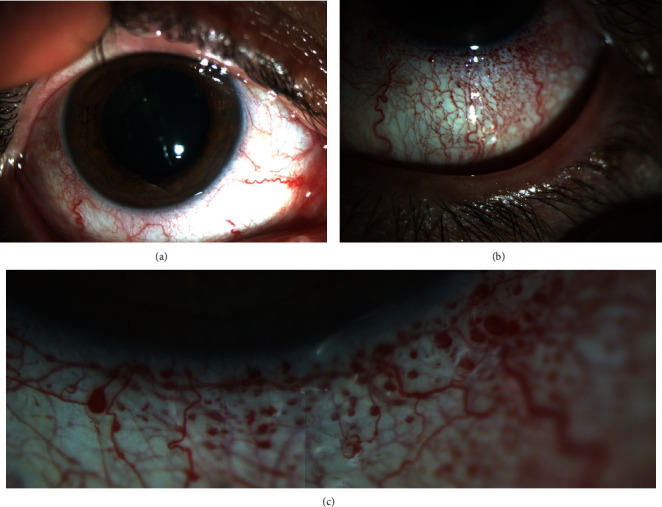
(a) Anterior segment picture of the right eye depicting normal conjunctival and episcleral vessels. (b, c) Anterior segment picture of the left eye depicting the conjunctival and episcleral vascular dilation, tortuosity, and conjunctival telangiectasia.

**Figure 2:**
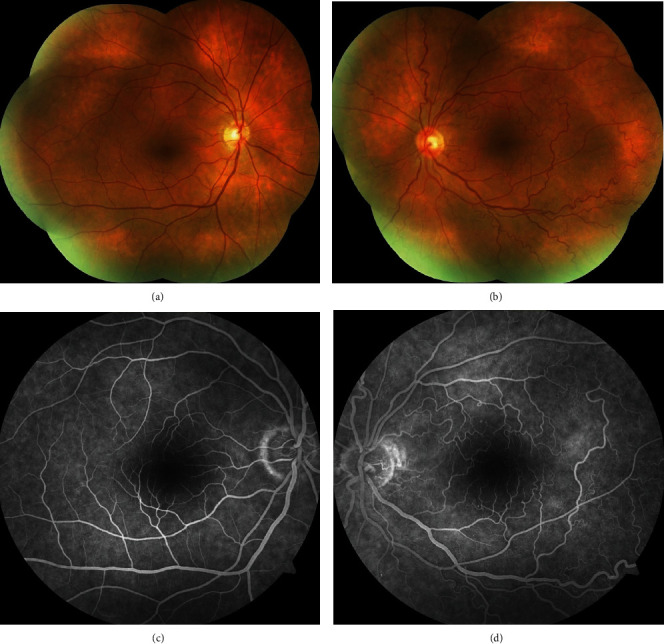
Colour fundus photograph of the right (a) and the left (b) eyes. Fundus fluorescein angiography of the right (c) and the left (d) eyes, showing normal retinal vasculature on the right and the convoluted and slightly dilated arteries and veins on the left eye.

**Figure 3:**
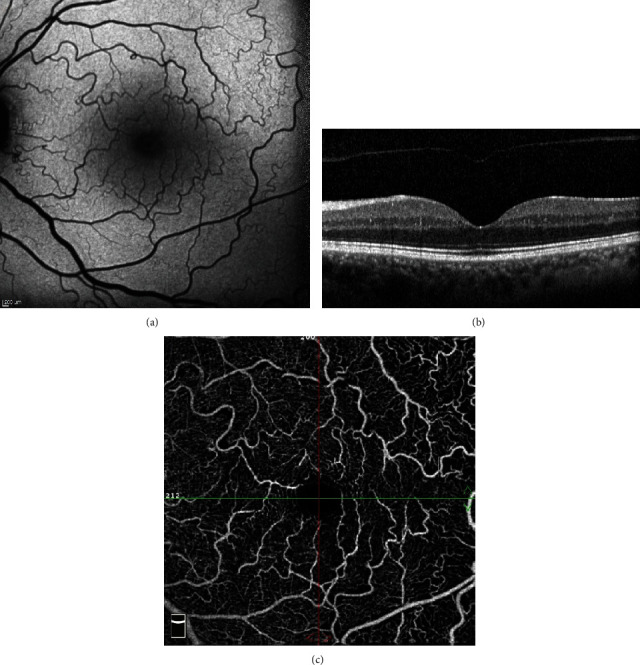
Fundus autofluorescence (a), optical coherence tomography (b), and optical coherence tomography angiography (c) imaging of the left eye.

Static and dynamic assessment of both eyelids were normal without any port-wine stain, hemangioma, or facial abnormality. There was no exophthalmos or globe deviation. On ophthalmological examination, the best-corrected visual acuity was 20/20 in both eyes; intraocular pressure was 16 mmHg in the right and 17 mmHg in the left eye. Slit-lamp anterior segment examination of the right eye was normal; however, left eye revealed conjunctival and episcleral vascular dilation and tortuosity with an accompanying bulbar conjunctival telangiectasia most prominent in the lower conjunctiva near to limbus (Figures 1(a)–1(c)). The dilated examination of the fundus and fluorescein angiography was normal in the right eye; contrary, the retinal vessels were strikingly tortuous and slightly dilated in all four quadrants of the left eye without any other accompanying retinal findings such as exudation or hemorrhage (Figures 2(a)–2(d)). The optic disc and macula were normal bilaterally. There was no mass lesion, retinal capillary haemangioma, choroidal haemangioma, or racemose angioma in the retinal periphery on indirect ophthalmoscopy. Fundus autofluorescence imaging and optical coherence tomography angiography of the left eye revealed a striking retinal vascular tortuosity; optical coherence tomography imaging scans were normal for both eyes (Figures 3(a)–3(c)). Schirmer I and tear break-up time tests were within normal limits bilaterally.

The patient was referred to internal medicine, cardiology, dermatology, and neurology departments in order to exclude some systemic syndromes that can accompany conjunctival telangiectasia such as hereditary hemorrhagic telangiectasia (Rendu-Osler-Weber disease), ataxia telangiectasia, Fabry's disease, Alport syndrome, and Bloom syndrome and some systemic diseases that can accompany retinal vascular tortuosity such as systemic hypertension, pulmonary arterial hypertension, atherosclerosis, polycythemia vera, and sickle cell anemia. Neuroradiological assessment including brain and orbital magnetic resonance imaging with magnetic resonance angiography was performed in order to exclude any accompanying retroorbital or cranial vascular abnormality and caroticocavernous fistula. After all those aforementioned evaluations, no systemic coexistence was identified.

The patient was monitored for visual acuity, intraocular pressure, slit-lamp anterior segment, and fundus examinations during follow-up visits. The clinical findings were stable without any symptoms, and there was no change in the appearance and shape of the conjunctival, episcleral, and retinal vessels 12 months after initial examination.

## 3. Discussion

We herein report an extremely rare ocular abnormality of isolated unilateral bulbar conjunctival telangiectasia and ipsilateral retinal vascular tortuosity. Despite thorough systemic evaluation and imaging, we could not able to demonstrate any systemic or neuroradiological association, and ocular findings were stable during a 1-year follow-up duration.

The most commonly encountered and well-defined systemic syndromes that can accompany conjunctival telangiectasia are hereditary hemorrhagic telangiectasia (Rendu-Osler-Weber disease) and ataxia telangiectasia, and rarely Alport syndrome, Fabry's disease, and Bloom syndrome.

Hereditary hemorrhagic telangiectasia (Rendu-Osler-Weber disease) is characterized by bleeding telangiectasias throughout various vascular beds in the body. Eye involvement occurs in 45-65% of patients, and the most common lesions are conjunctival telangiectasias in the palpebral conjunctiva [4]. There are also some reports in the literature defining hemolacria as a finding of hereditary hemorrhagic telangiectasia [4–6]. Unilateral and bilateral conjunctival involvement has been reported in the literature. Dilatation of retinal vessels due to retinal arteriovenous malformation has been rarely seen [4, 5]. So, it was also considered in the differential diagnosis of our patient. Diagnosis of the disease is based on clinical findings of mucosal and/or skin lesions and bleeding from the lesions, and a family history [4]. However, the patient in this case had no recurrent epistaxis, hemolacria, muco-cutaneous telangiectasias, or family history of the disease. Choroidal telangiectasia was also reported in a patient with hereditary hemorrhagic telangiectasia by using indocyanine green (ICG) angiography [7]. Although retinal arteriovenous malformation was excluded by using fundus fluorescein angiography, we could not completely exclude the possibility of the presence of choroidal telangiectasia, since we did not perform ICG angiography.

Ataxia telangiectasia is an autosomal recessive disorder primarily characterized by immunodeficiency, radiation sensitivity, and cancer susceptibility along with some neurological findings such as cerebellar degeneration [8]. In ataxia telangiectasia, conjunctival-cutaneous telangiectasia are the main ocular findings. However, retinal telangiectasia or retinal vascular changes are not evident [9]. Our patient is an otherwise healthy adult which also excludes the possibility of this diagnosis.

Conjunctival and retinal vascular tortuosity and dilatation are common in Fabry's disease. However, these often present bilaterally and vascular involvement that is mostly combined with vortex keratopathy (cornea verticillata) and lens opacity with a “spoke-like” pattern at the posterior capsule in Fabry disease [10]. These findings correlate with progressive deposition of glycosphingolipids in the ocular structures.

Caroticocavernous fistula should also be kept in mind in a patient with unilateral conjunctival and retinal vascular dilatation and tortuosity, which is an abnormal connection between the cavernous sinus and the carotid artery system and usually occurs unilaterally. These patients may have signs and symptoms such as corkscrew episcleral blood vessels in association with conjunctival chemosis and dilated, tortuous retinal veins with retinal hemorrhage, as well as proptosis, pulsating exophthalmos, diplopia, ophthalmoplegia, elevated intraocular pressure, and audible bruits [11, 12]. The patient was referred to a neuroradiology unit in order to exclude caroticocavernous fistula in the process of differential diagnosis; however, brain and orbital magnetic resonance imaging along with magnetic resonance angiography scans, all were normal.

Familial retinal arterial tortuosity is another rare disease that should be kept in mind in differential diagnosis of retinal vascular tortuosity, which is an autosomal dominant disorder affecting the retinal arterioles rather than the venous system [13]. In some cases, it has been reported that the retinal arteriolar tortuosity is accompanied by bulbar conjunctival telangiectasia [14]. Retinal arterioles typically appear in corkscrew and spiral shapes and can lead to recurrent visual symptoms complicated by preretinal and intraretinal hemorrhages [15]. Systemic involvement of nonocular vascular beds has been demonstrated in a few cases [16]. However, the diagnosis is mainly based on clinical findings of bilateral tortuosity of small and medium-sized arterioles in the peripapillary and macular regions contrary to our patient which has unilateral involvement.

Impending retinal vein occlusion can also be considered in differential diagnosis of unilateral retinal venous dilatation and tortuosity. However, both retinal and venous systems were involved in our patient. There is also no defined association of impending vein occlusion with conjunctival telangiectasia.

Last but not least, some acquired causes such as chronic phases of some ocular surface disorders should also be considered in the differential diagnosis of conjunctival telangiectasia [3, 17, 18]. Of these, ocular rosacea is the mostly encountered one. However, unilateral involvement in our patient and absence of symptoms such as foreign body sensation and burning; absence of ocular findings such as meibomian gland dysfunction, blepharitis, lid margin erythema and irregularities, and tarsal conjunctival telangiectasia helped us to exclude acquired causes in differential diagnosis.

In conclusion, isolated unilateral bulbar conjunctival telangiectasia and ipsilateral retinal vascular tortuosity without any systemic or neuroradiological association is an extremely rare coexistence. A detailed and thorough systemic evaluation is necessary to exclude some severe diseases as the underlying cause in these patients.

## References

[1] Decock C., De Laey J. J., Leroy B. P., Kestelyn P. H. (2003). Alport syndrome and conjunctival telangiectasia. *Bulletin de la Société Belge d'Ophtalmologie*.

[2] Sahn E. E., Hussey R. H., Christmann L. M. (1997). A Case of Bloom Syndrome with Conjunctival Telangiectasia. *Pediatric Dermatology*.

[3] Müftüoğlu İ. K., Akova Y. A. (2016). Clinical Findings, Follow-up and Treatment Results in Patients with Ocular Rosacea. *Türk Oftalmoloji Dergisi*.

[4] Knox F. A., Frazer D. G. (2004). Ophthalmic presentation of hereditary haemorrhagic telangiectasia. *Eye (London, England)*.

[5] Brant A. M., Schachat A. P., White R. I. (1989). Ocular manifestations in hereditary hemorrhagic telangiectasia (Rendu-Osler-Weber disease). *American Journal of Ophthalmology*.

[6] Rinaldi M., Buscarini E., Danesino C. (2010). Ocular manifestations in hereditary hemorrhagic telangiectasia (Rendu-Osler-Weber disease): A case-series. *Ophthalmic Genetics*.

[7] Tsai D. C., Wang A. G., Lee A. F., Hsu W. M., Liu J. H., Yen M. Y. (2002). Choroidal telangiectasia in a patient with hereditary hemorrhagic telangiectasia. *Eye (London, England)*.

[8] Lavin M. F. (2008). Erratum: Ataxia-telangiectasia: from a rare disorder to a paradigm for cell signalling and cancer. *Nature Reviews Molecular Cell Biology*.

[9] Farr A. K., Shalev B., Crawford T. O., Lederman H. M., Winkelstein J. A., Repka M. X. (2002). Ocular manifestations of ataxia-telangiectasia. *American Journal of Ophthalmology*.

[10] Morier A. M., Minteer J., Tyszko R., McCann R., Clarke M. V., Browning M. F. (2010). Ocular manifestations of Fabry disease within in a single kindred. *Optometry*.

[11] Zhu L., Liu B., Zhong J. (2018). Post-traumatic right carotid-cavernous fistula resulting in symptoms in the contralateral eye: a case report and literature review. *BMC Ophthalmology*.

[12] Chaudhry I. A., Elkhamry S. M., Al-Rashed W., Bosley T. M. (2009). Carotid cavernous fistula: ophthalmological implications. *Middle East African Journal of Ophthalmology*.

[13] Sutter F. K. P., Helbig H. (2003). Familial Retinal Arteriolar Tortuosity: A Review. *Survey of Ophthalmology*.

[14] Seo J. H., Kim I., Yu H. G. (2009). A case of carotid aneurysm in familial retinal arterial tortuosity. *Korean Journal of Ophthalmology*.

[15] Gekeler F., Shinoda K., Jünger M., Bartz-Schmidt K. U., Gelisken F. (2006). Familial retinal arterial tortuosity associated with tortuosity in nail bed capillaries. *Archives of Ophthalmology*.

[16] Saraf S. S., Tyring A. J., Chen C. L. (2019). Familial retinal arteriolar tortuosity and quantification of vascular tortuosity using swept-source optical coherence tomography angiography. *Am J Ophthalmol Case Rep.*.

[17] Akpek E. K., Merchant A., Pinar V., Foster C. S. (1997). Ocular Rosacea. *Ophthalmology*.

[18] De Marchi S. U., Cecchin E., De Marchi S. (2014). Ocular rosacea: an underdiagnosed cause of relapsing conjunctivitis-blepharitis in the elderly. *Case Reports*.

